# Searching for Chaos Evidence in Eye Movement Signals

**DOI:** 10.3390/e20010032

**Published:** 2018-01-07

**Authors:** Katarzyna Harezlak, Pawel Kasprowski

**Affiliations:** Institute of Informatics, Silesian University of Technology, 44-100 Gliwice, Poland

**Keywords:** eye movement, nonlinear time series analysis, chaotic behaviour

## Abstract

Most naturally-occurring physical phenomena are examples of nonlinear dynamic systems, the functioning of which attracts many researchers seeking to unveil their nature. The research presented in this paper is aimed at exploring eye movement dynamic features in terms of the existence of chaotic nature. Nonlinear time series analysis methods were used for this purpose. Two time series features were studied: fractal dimension and entropy, by utilising the embedding theory. The methods were applied to the data collected during the experiment with “jumping point” stimulus. Eye movements were registered by means of the Jazz-novo eye tracker. One thousand three hundred and ninety two (1392) time series were defined, based on the horizontal velocity of eye movements registered during imposed, prolonged fixations. In order to conduct detailed analysis of the signal and identify differences contributing to the observed patterns of behaviour in time scale, fractal dimension and entropy were evaluated in various time series intervals. The influence of the noise contained in the data and the impact of the utilized filter on the obtained results were also studied. The low pass filter was used for the purpose of noise reduction with a 50 Hz cut-off frequency, estimated by means of the Fourier transform and all concerned methods were applied to time series before and after noise reduction. These studies provided some premises, which allow perceiving eye movements as observed chaotic data: characteristic of a space-time separation plot, low and non-integer time series dimension, and the time series entropy characteristic for chaotic systems.

## 1. Introduction

Most naturally-occurring physical phenomena are examples of nonlinear dynamic systems, the functioning of which attracts many researchers seeking to unveil their nature and gradual development. The main obstacle in the exploration of a physical system’s dynamics is the lack of directly obtainable formulas defining a system’s behaviour, thereupon specific patterns have to be inferred from observations of system states usually represented by a sequence of scalar measurements. According to the nonlinear dynamic system theory, it is feasible to reconstruct a nonlinear system and the path it follows, while evolving in time, if given a time series obtained from one of the system’s variables. This is possible due to usage of the embedding theorem, which provides an association between the theory of nonlinear dynamical systems and the analysis of experimental time series by embedding observations with time delay.

This approach has been already adapted in many branches, such as physics, astrophysics, chemistry, economics and biology. Recently, it has also become increasingly useful in enhancing knowledge of human biological systems. A comprehensive review of nonlinear system analysis in this field was provided in [1]. Some other works focused on the usage of different nonlinear methods in cardiology to characterise the heart rate in various physiological and pathological conditions [2,3] and to provide an improvement in heart disease diagnostics. The heart blood vessel system was investigated in terms of nonlinear dynamic behaviour under certain parameters by the use of various methods for fractal dimension and entropy evaluation [2]; and by the analysis of the phase portrait, power spectra, bifurcation diagram and Poincaré map [4]. The authors of [5,6] research studied the embedding theory to examine variability in human movement, stride-to-stride fluctuations, and the influence of local instabilities on the global gait cycle. Many studies may also be encountered in the field of neuroscience such as a review of the data and main arguments which support the existence of chaos in the behaviour of the nervous system [7]; or studies concerning chaotic measures in regard to Electroencephalography signals (EEG) [8].

The research presented in this manuscript is devoted to analysis of eye movements—the biological signal produced by the oculomotor system responsible for managing twelve muscles by means of neural signals carrying information regarding eye position and velocity. The direction of the movement is achieved by activating the appropriate eye muscles, whereas the eye movement amplitude is dependent on the duration of neuronal activity in the lower motor neurons of the oculomotor nuclei [9].

### 1.1. Eye Movement Basis 

Eye movement signals may be registered by means of specialised devices called eye trackers, which may utilise different techniques for this purpose. The most common approach is video-based eye tracking (Video-oculography, VOG), which records eye movement by means of digital video cameras. The eye positions and movements are determined based on the information acquired from the sequence of registered images. VOG eye trackers may be of various types: head-mounted (e.g., glasses), tower-mounted with a chin rest, or remote. The most important device feature, from the current research point of view, is its sampling rate. Eye movements are incredibly fast—their speed may reach 800 deg/s—thus to reveal eye movement characteristics as complete as possible, it is desirable to record at least 500–1000 samples per second. Additionally, an appropriate sampling rate provides a greater set of measurements, which may improve the accuracy of the data analysis methods applied. 

The construction of the eye and the way of its functioning induce the existence of two main components in eye movement: (1) a fixation, during which the eye is almost motionless and acquires information from an observed scene; (2) a saccade—a quick movement between fixations during which no information is taken. When analysing the first of the components, some very small movements may be found, namely: tremors (aperiodic, wave–like motion), microsaccades (small, fast movements during voluntary fixation) and drifts (slow motions of the eye that occur between microsaccades) [10,11].

Many studies have been undertaken in order to understand and describe the functioning of the eye. There are several works defining models for systems controlling eye movements: models of the saccadic eye movement as presented in [12]; the oculomotor plant mathematical model shown in [13] and the model of fixational eye movements and microsaccades described in [14] are only a few of the selected examples. Additionally, studies aimed at understanding the relationship between neural activity and eye movements were conducted [9,15,16].

Yet, the exploration of eye movement dynamics with the usage of methods for nonlinear system analysis has been addressed only in several studies. In [17,18] Aştefănoaei et al. examined the trend in saccadic movement based on a visually-guided saccade task and one with a cognitive load. One healthy adult male volunteer took part in both experiments. The time series data used in the research consisted of x- and y-directional eye-gaze locations. In the conducted analysis, signal power spectrum, and the Lapunov and Hurst exponents were taken into account. The methods applied confirmed the presence of high complexity dynamics representing deterministic chaotic behaviour, increasing in complexity with task difficulty.

The purpose of another study [19] was to explore eye movements, by the application of nonlinear dynamics (chaos analysis), while a visual search task, with changing layout complexity, was realized. Similarly, the first Lyapunov exponent and the attractor plot were determined for the time series data of x- and y-directional eye-gaze locations. The results showed that the first Lyapunov exponent exhibited positive values, and tended to increase when a more difficult task was performed. The same concerned the attractor plot, whose complexity increased in line with the more complicated tasks.

Eye functioning in terms of chaotic behaviour was also studied in [20] in regard to the accommodative process invoked in order to change the focus on an object as its distance varies. The influence of reflective error and the changing object’s distance on fluctuations in relation to the power of the lens were analysed. Tests performed did not reveal any effect of accommodative demand on the Lyapunov Exponent, embedding lag and embedding dimension, while they presented such an influence in the case of reflective error on the Lyapunov Exponent and embedding dimension. 

There are also two works exploring chaotic behaviour in eye movements based on the first derivate for horizontal and vertical eye positions during imposed, prolonged fixations. The first Lyapunov exponent was analysed within several fixation’s time intervals. Its analysis revealed chaotic behaviour in eye movements just after a stimulus—a “jumping point”—position change. Subsequently, signals were changing behaviour from convergent to chaotic and conversely [21,22].

### 1.2. Contribution

The research presented in this paper is the continuation of the two previously-mentioned studies aimed at exploring other eye movement features and confirming the findings of those studies. For this purpose, the same time series were used, however different methods of nonlinear system analysis—correlation dimension and signal entropy—were applied. Several aspects were taken into consideration for both quantities’ evaluation and the contribution of the research is as follows:

1. The analysis of eye movement signal in terms of chaotic features existence in the time series defined based on horizontal eye movement velocity:
  - evaluation of the correlation dimension for various length of the measurement sets;
  - estimation of the entropy for different time intervals of the fixation duration;
  - analysis of the temporal correlation existence in the sets of measurement.
2. The comparative analysis of data before and after noise reduction.
3. The comparative analysis of the obtained results and characteristics of the Largest Lyapunov Exponent.

## 2. Theory

An observation of a biological system produces a *N*-element time series obtained at time intervals *T*:

*x*(*t*) *=* {*x*(*t*_0_), *x*(*t*_1_), …, *x*(*t_k_*), …, *x*(*t_N_*)} (1)

The reconstruction of this system phase space, according to the embedding theory, requires the scalar sequence of measurements *x*(*t*) to be replaced with data vectors *y*(*i*) in a time delay Euclidean *m*-dimensional space [23]:

*y*(*i*) *=* {*x*(*i*), *x*(*i +τ*), *x*(*i +* 2 *×τ*), …, *x*(*i +* (*m − 1*) *×τ*)} (2)

where *τ* is called time lag and is a multiplicity of *T* and *i =* {1, 2, …, *M*}, where *M = N −* (*m −* 1) *× τ*. The dimension *m* of the time delay phase space, constructed based on the set of vectors *y*(*i*), need not be the same as the dimension of the underlying system. Even then, the time delay phase space preserves all invariants of the original system motion. However, a crucial issue is proper reconstruction of the phase space and the choice of its parameters, namely: time lag *τ* and embedding dimension *m*.

1. Time lag—the most common approach, adopted for this purpose, is the evaluation of mutual information between measurements calculated in the form of the *averaged mutual information factor* (Equation (3)):
  (3) $$I\left(T\right)=\overset{j}{\underset{h=1}{\sum }}\overset{j}{\underset{k=1}{\sum }}P_{h,k}\left(\tau \right)\log_{2}\frac{P_{h,k}\left(\tau \right)}{P_{h,}P_{k}}$$
  where *P_h_* and *P_k_* denote the probabilities that the signal assumes a value within the *h*-th and *k*-th intervals respectively, and *P_h,k_*(*τ*) is the joint probability that *x_i_* is taken from interval *h* and *x_i_ + τ* from interval *k* [24].
2. Embedding dimension—for estimating the second of the aforementioned parameters the *False Nearest Neighbours* (FNN) method proposed in [25] is used. According to this method, *y_i_^NN^* is the nearest neighbour of *y_i_* when the ratio of distances between these points seen in the dimension *m* + 1 and in dimension *m*, is less than the predefined threshold *R*—Equation (4):
  (4) $$R=\frac{\left|x_{i+\left(m+1\right)\times \tau }-x_{i+\left(m+1\right)\times \tau }^{NN}\right|}{\left\|y_{i}-y_{i}^{NN}\right\|}$$
  where $x_{i+\left(m+1\right)\times \tau }$ and $x_{i+\left(m+1\right)\times \tau }^{NN}$ are additional components of vector *y*(*i*) in *m +* 1 dimensional space and *y_i_* and *y_i_^NN^* are vectors of *m* coordinates. According to Takens’ theorem [26], a system attractor can be restored correctly, if the embedding dimension *m* satisfies the condition: *m* ≥ *2d* + 1, where *d* represents the dimension of an attractor.
  The estimation of phase space parameters enables further analysis aimed at system classification and identification based on analysis of phase space trajectories. Deterministic dynamical systems evolving typically towards an *attractor*, are characterized by a dimension less than the full phase space, whereas random time series are supposed to fill the embedding space. In order to quantify these occurrences, fractal or chaos methods, in the form of the fractal dimension and Lyapunov Exponents respectively, may be applied.
3. Largest Lyapunov Exponents—two similar algorithms were proposed by Kantz [27] and Rosenstein [28] for calculating this exponent as:
  (5) $$ʎ=\frac{1}{\Delta t}\ln\left(\frac{d_{j}}{d_{j0}}\right)$$
  where *d_j_* is the Euclidean distance between a *j*-th pair of the nearest neighbours after time steps *∆t = t_i_ − t_0_* and *d_j0_* is the initial separation of neighbours in a considered pair.
4. Fractal Dimension—although, there are several algorithms for ascertaining the fractal dimension, the method most commonly used is the correlation dimension first introduced by Grassberger and Procaccia [29]. It is based on a correlation sum being a collection of a fraction, or all possible pairs of points, which are closer than a particular distance *r* (Equations (6) and (7)):
  (6) $$C\left(r\right)=\frac{2}{N\left(N-1\right)}\overset{N}{\underset{i=1}{\sum }}\overset{N}{\underset{j=i+1}{\sum }}H\left(r-\left\|x_{i}-x_{j}\right\|\right)$$
  where *H* is the Heaviside function defined as follows:
  (7) $$H\left(x\right)=\{\begin{array}{c} 0,\;x>r\\ 1,\;x\leq r \end{array}$$
  The correlation dimension of the dynamic system attractor is defined as the limit:
  (8) $$D_{2}=\lim_{N\to \infty \;r\to 0}\left(\frac{\log C\left(r\right)}{\log r}\right)$$
  - Estimation of required measurements number ‒ according to [30] the upper limit on a reliable estimation of *D_2_* is feasible when the number *N* of data points in the time series is given by:
    (9) $$N\geq 10^{\frac{D_{2}}{2}}$$
    When reversing the question and asking: what is a correlation dimension, which can be reliably calculated with *N* samples, we obtain:
    (10) $$D_{2}\leq 2\log_{10}N$$
  - Temporal Correlation—to avoid a problem of a temporal correlation present in measurements [31] the points to be considered in the correlation sum must be separated by *tmin* = *w × ∆t*, where *w* is the number of points excluded from the calculation and is called the Theiler window [32]. This entails changes in the formula of *C*(*r*):
    (11) $$C\left(r\right)=\frac{2}{\left(N-w\right)\left(N-w-1\right)}\overset{N}{\underset{i=1}{\sum }}\overset{N}{\underset{j=i+1+w}{\sum }}H\left(r-\left\|x_{i}-x_{j}\right\|\right)$$
    The Theiler window may be determined by multiplying the earlier estimated time lag by three or by analysis of the space-time separation plot, introduced by [33], which shows lines of constant probability for distribution of distances between pairs of points in function of time.
5. Entropy—sensitivity of chaotic systems to initial conditions introduces some uncertainty in measurements. The rate of new information introduced during system evolvement may be assessed by the correlation entropy *K_2_* defined—based on the correlation sum *C(m, r)* evaluated for sets of embedding dimensions *m* and radiuses *r*—as [31]:
  (12) $$K_{2}=\underset{r\to 0}{\lim}\;\underset{m\to \infty }{\lim}\;\underset{n\to \infty }{\lim}\frac{C\left(m,\;r\right)}{C\left(m+1,r\right)}$$

## 3. Materials and Methods 

**Figure 1:**
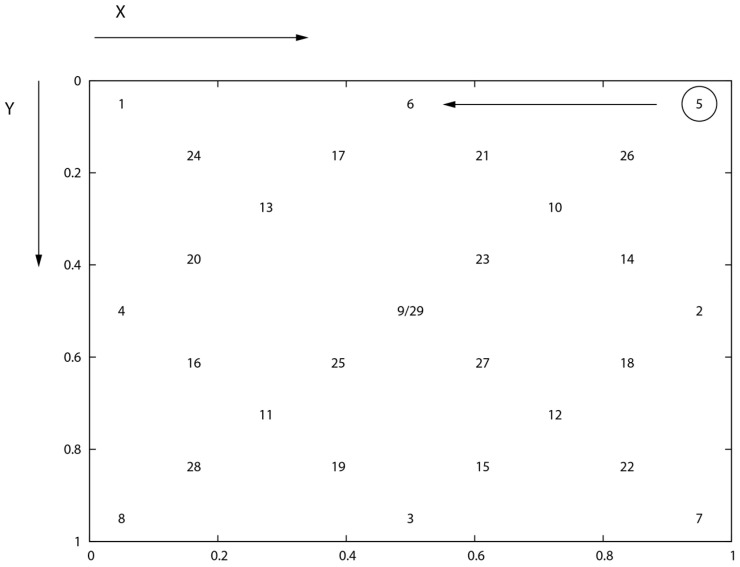
Layout of stimuli applied during the experiments—based on the universal scale—the top-left corner of the screen is represented by (0.0, 0.0) coordinates and the bottom-right corner by (1.0, 1.0) respectively. An example direction of a stimulus movement from one location (5) to another (6) was presented [21].

The analysis of eye movement characteristics was conducted with the usage of a data set collected during the experiment based on a “jumping point” represented by a dark dot of size 0.5 × 0.5. This dot was shown in 29 various locations distributed over a white 1280 × 1024 (370 mm × 295 mm) flat screen. Coordinates of each location were defined in the universal scale where the (0.0, 0.0) point is located at the top–left–hand corner and (1.0, 1.0) one at the bottom–right–hand corner of the screen (Figure 1). A point position, expressed in degrees of visual angle or in a metric system, may be calculated based on the screen size and eye–screen distance. In the case of the presented studies, the horizontal and vertical visual angles amounted to approximately 40° and 32°, while the eye–screen distance was equal to 450 mm. Each point was displayed for 3000 ms, during which the point size was changing in order to facilitate the keeping of users’ concentration.

Twenty four people—male and female students with normal vision, aged between 22 and 24—took part in the experiment, which consisted of two sessions separated by a two-month interval. Before each experiment, the participants were informed of the general purpose of the experiment, after which they signed a consent form. Each participant’s session started with a calibration process and only those trials with calibration errors lower than 1 degree were subjected to further processing. Data from approximately 10% of trials was subject to rejection. 

For collecting data, a head-mounted Jazz–Novo eye tracker (product by Ober–Consulting [34], Poznan, Poland)—capable of recording eye positions with a 1000 Hz sampling rate—was used. It is based on Infra–Red Oculography (IROG) and is equipped with pairs of IR emitters and sensors. From the hardware and software point of view, a computer with Windows 7, Intel Xeon w3550 CPU, 3.07 GHz, 8.00 GB RAM and 64 bits operational system was used.

The experimental dataset comprised of 48 signal records, within which 29 fixations, consisting of 3000 samples, may be distinguished. An eye movement signal, collected for each out of the 29 stimulus locations, was utilised for defining time series. For this purpose the standard procedure of the two–point signal differentiation was used to calculate the first derivative of the horizontal positions of eye movement. It provided 48 × 29 = 1392 time series, which were further explored with the usage of methods for time series analysis available in R language. Taking the sampling rate into account, which amounted to 1000 Hz and the time of stimulus presentation—3000 ms—in each time series 2999 measurements were available. 

**Figure 2:**
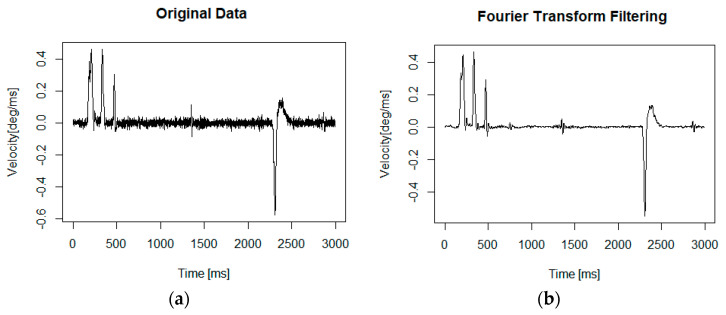
An example time series (**a**) in original form (BNR) (**b**) after noise reduction (ANR). The horizontal axis is represented in ms, the vertical in degrees of visual angle per ms. Another example of the ideal low–pass filter application may be found in [21].

The problem of noise, always present in measured time series [35], possibly resulting from environmental and measurement errors [36], as well as introduced by the oculomotor system, was addressed before each time series’ examination. Previous research conducted by means of the same device used in this study revealed the existence of linear components in registered signals [37]. Thus, for the purpose of noise elimination, registered signals were analysed in the frequency domain. Based on the Discrete Fourier Transform, the ideal low–pass filter with a 50 Hz cut–off frequency was applied (Figure 2). However, both time series types, before and after noise reduction—further referred to as BNR (before noise reduction) and ANR (after noise reduction) respectively—were subjected to subsequent analysis.

## 4. Results

One of the fundamental problems present in the analysis of signals obtained through an experiment is how to assess the influence of the noise contained in the data. On the other hand, it is interesting what impact has the utilized filter on the obtained results. In order to explore this effect, all further concerned methods were applied to time series before and after noise reduction. 

**Figure 3:**
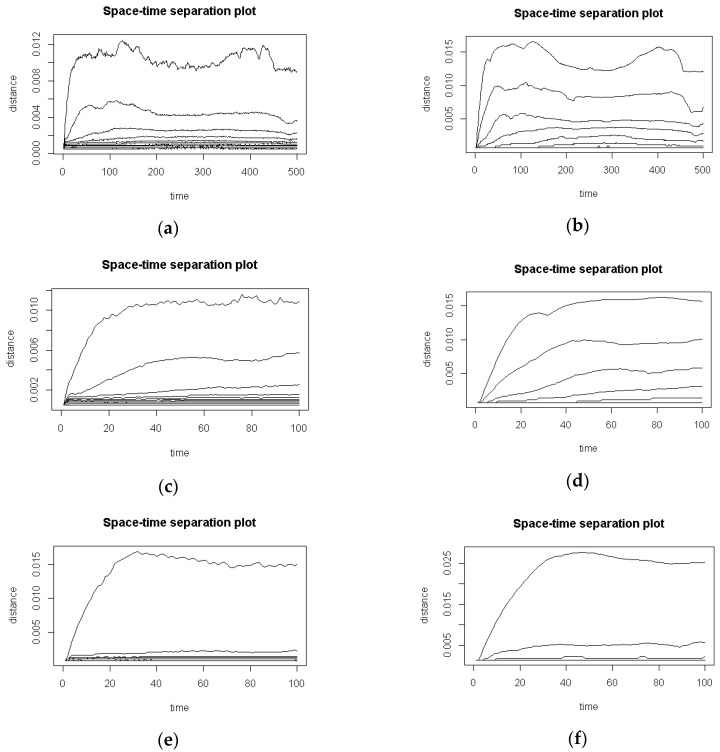
Space-time separation plots: (**a**) an example of BNR time series; *τ* = 3 and *m* = 3; (**b**) the same time series after noise reduction ANR; *τ* = 9 and *m* = 4 (**c**) the same BNR time series in a shorter time perspective; (**d**) the same ANR time series in a shorter time perspective; (**e**,**f**) another time series BNR and ANR, with different saturation time. For all cases, a Theiler window equal to 100 was chosen.

Two issues were considered at the beginning of the time series analysis. At first, both their types were explored in terms of temporal correlation by examination of space-time separation plots obtained with usage of the *stplot* function from the tseriesChaos package [38]. Their visual inspection revealed that the safe temporal separation for defining a *Theiler window* equals to 100. Figure 3 presents examples of space-time separation plots acquired for the same time series, before and after noise reduction—plots Figure 3a,b respectively and—visible in a shorter time perspective—Figure 3c,d. In the same time period another time series BNR and ANR was shown on plots Figure 3e,f to illustrate some differences in the saturation of contour lines. These differences may be noticed (1) among BNR and ANR times series—Figure 3c,d and Figure 3e,f—on the left-hand plots, lines saturate slightly quicker; and (2) among the same time series types BNR (Figure 3c,e) and ANR (Figure 3d,f).

Time lag for each time series—BNR and ANR—was evaluated individually with usage of the *mutual* function, while the embedding dimension by means of the *false.nearest* one, both from the tseriesChaos package [38]. The number of bins—the *mutual* function parameter—was determined by usage of the Freedman and Diaconis method. 

Another concern, addressed during data pre-processing, was related to the finite size of a measurements set and its usability in the current studies. Each analysed time series consisted of 2999 elements. Substituting this value into Equation (10), we may learn that such a number of measurements ensures safe estimation of the correlation dimension equal to or less than seven.

Furthermore, the maximal possible correlation dimension for a set containing 1000 and 1500 measurements was checked as, in other “jumping point” applications, a stimulus may be presented shorter than 3000 ms [39]. This also regards the calibration process conducted before each eye tracking session, during which people are expected to follow with their eyes, a point changing its position on a screen [37,40]. Many manufactures incorporate such a procedure into their eye tracker setup. The results obtained for sets of measurements varying in length are presented in Table 1.

### 4.1. Correlation Dimension Exlploration

Correlation dimension was assessed for each defined time series—BNR and ANR—by means of the *corrDim* function available in the nonlinearTseries package [41]. The parameters for defining vectors in this phase space—time lag *τ* and embedding dimension *m*—were the input data for the *corrDim* function. Time lag was evaluated for each time series independently, with usage of the *mutual* function, while the *embedding dimension* ranged from 3 to 15. Additionally, the *corrDim* function takes two parameters determining the scope of analysed radiuses, which were calculated as:

(13) $$rmin=\frac{stdev\left(time\;series\;values\right)}{10}\;rmax=10\times stdev\left(time\;series\;values\right)$$

**Figure 4:**
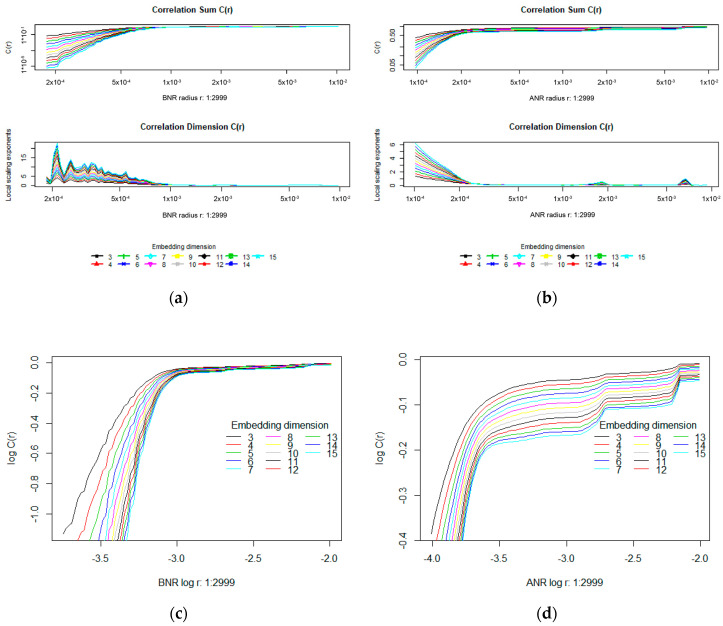
Plots of the correlation sum and local scaling exponent in function of radius *r* for the same time series before and after noise reduction—panels (**a**,**b**) respectively. Panels (**c**,**d**) present log(*c*(*r*)) vs. log(*r*) plots for the same time series.

The next two required parameters, the number of radiuses analysed in the range of (*rmin*, *rmax*) and the Theiler window, were set to 100. Plots of correlation sums, scaling exponents and log(*C*(*r*)) vs. log(*r*) for a sample time series, before and after noise reduction, are presented in Figure 4. The two first mentioned plots are visible in Figure 4a,b on the top and at the bottom of the panels, respectively, while log-log charts are shown in Figure 4c,d. The left-hand part of Figure 4 is dedicated to the BNR time series. On the right, the plots related to the ANR time series are shown.

Based on the presented plots it may be observed that for time series before noise reduction (BNR) the correlation sum values are lower than for the ANR sets when shorter radiuses are taken into account. For example, the correlation sum *C_BNR_* (*r*), within a radius equal to 0.0002, depending on the embedding dimension *m*, amounts to between 0.00001 and 0.1 approximately, while for the ANR set it assumes roughly a value of 0.5 for all embedding dimensions. Similarly, a saturation in the correlation sum for the BNR time series was obtained for a longer radius (>0.0005) than in the case of the ANR one (around 0.0002). 

**Figure 5:**
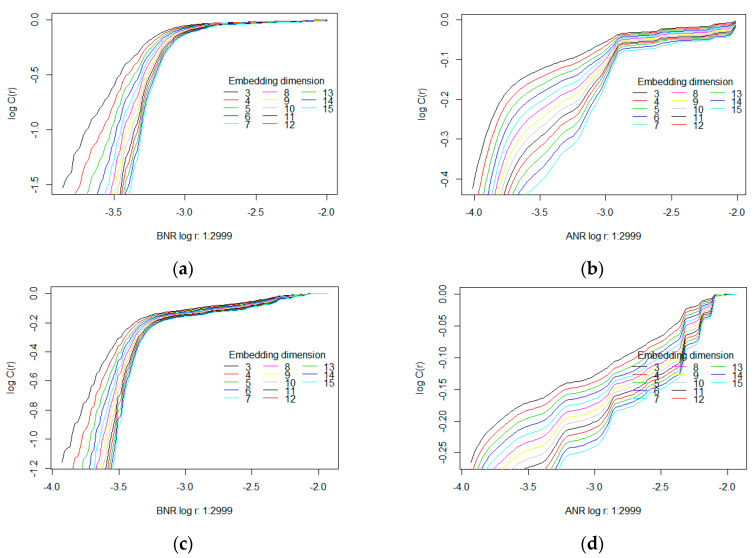
log(*C*(*r*)) vs. log(*r*) plots for BNR (**a**,**c**) and ANR (**b**,**d**) time series for two different people. Panel (**a**,**b**) correspond to the same person, whereas panels (**c**,**d**) to the second one.

Plots collected for various time series may vary among each other and some such differences are provided in Figure 5. The charts placed in the same row correspond to the same times series, however, the time series in different rows were defined based on eye movement signals belonging to different people.

When analysing log(*C*(*r*)) vs. log(*r*) plots, it may be noticed that in the case of the time series before noise reduction (the left-hand panels) they are similar in shape, while for the corresponding ANR charts, the differences may be easily perceived. However, the same as the above-mentioned dependency in the correlation sum values is still visible. It indicates that after removing signal frequencies over 50 Hz, the number of neighbours in the ANR time series increased within shorter radiuses. 

Another difference, which may be found between corresponding plots, is a slightly different characteristic after reaching saturation. The left-hand plots, after saturating, stay at the same level independently of an increase in radius length. The ANR time series reveal in turn some rapid growths in the correlation sum after several radius changes. In the case of the plot depicted in Figure 5d several such changes may be seen. 

The correlation dimension was estimated based on visual inspection of the degree of curve slope in log(*C*(*r*))-log(*r*) plots. If this graph shows an approximate linear dependence in some ranges of *r*, then the slope in this linear scaling range is the estimated correlation dimension. As the results for too low or too high values of *r* may be imprecise, scaling regions should be identified in the middle range of the curve. For the purpose of these studies the range of *r* was set to between:

- 0.0018 and 0.0055, giving log(*r*) between −2.74 and −3.26, for the BNR time series,
- 0.001 and 0.003, giving log(*r*) between −3 and −2.52 for the ANR time series.

The application of the one range for all time series may introduce some inaccuracy in slope approximations, yet acceptable in regards to rough estimation when low correlation dimensions are expected [42]. 

The described procedure was utilised for time series of different lengths—1000, 1500 and 2999 elements—in order to investigate the influence of a number of measurements on evaluating the correlation dimension. However, due to some calculation complexity which resulted from: (1) an insufficient number of neighbours; (2) performance of the phased space parameters evaluation; (3) the generalised scaling regions identification—the number of time series subjected to further processing had to be reduced from 1392 to 1250. By exploring each time series separately, the aforementioned problems could be easily solved by the adjusting methods’ parameters individually. 

**Figure 6:**
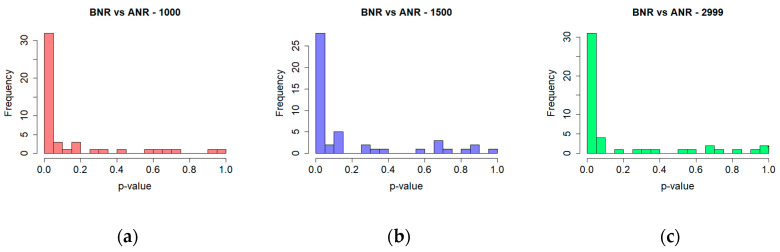
Significance of differences in the correlation dimension values for time series before noise reduction (BNR) and after noise reduction (ANR)—histogram of *p*-values for time series of length (**a**) 1000; (**b**) 1500; (**c**) 2999.

The correlation dimension values, regardless of log-log plots characteristics, turned out to be similar for the BNR and ANR time series (Table 2). In both cases values were lower than one and tended to decrease with an increase in the number of measurements. However, the differences were not substantial, thus the statistical significance of these differences was explored utilising the Wilcoxon test in regard to the BNR and ANR time series, as well as taking different time series lengths into consideration. This method choice was made based on the results of the Shapiro-Wilk test. It revealed that in the case of the majority of time series, the distributions of the correlation dimension values were not normal. The significance was evaluated for each of 48 analysed sessions and reported in the form of their percentage as well as in the form of a histogram of *p*-values (Figure 6). Results of the first test (BNR vs. ANR) calculated for the above-chosen lengths are provided in the last row of Table 2.

**Figure 7:**
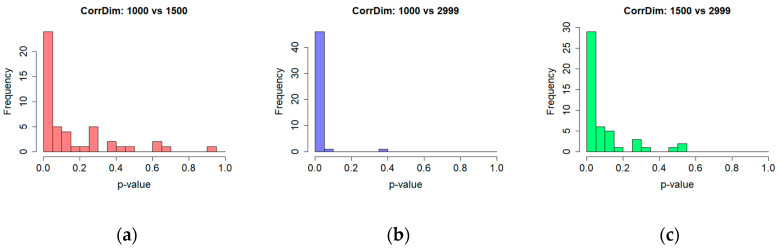
Significance of differences in the results obtained for the correlation dimension for different time series lengths—histogram of *p*-values for ANR time series of length (**a**) 1000 vs. 1500; (**b**) 1000 vs. 2999; (**c**) 1500 vs. 2999.

The outcomes of tests concerning time series’ length are shown in Table 3 and Figure 7. They revealed a relationship between the difference in the length of time series and the significance of differences in the obtained results: the greater the length difference the higher the percentage of significant differences. 

According to the earlier-conducted studies [43], applying filters to signals obtained through an experiment may have the impact on the correlation dimension evaluation by means of the Grassberger and Procaccia algorithm. In those studies the authors revealed that filters, when improperly applied to random signals, can mimic some chaotic behaviour.

In order to exclude such a possibility in the current research, the *corrDim* method was applied to surrogate data, as suggested by the authors of the paper. The surrogate sets—five sets for each time series—were prepared based on the original time series with usage of the *FFTsurogate* function from the nonlinearTseries package [41]. The obtained results—in the form of log-log plot—revealed that correlation dimensions did not converged for all analysed embedding dimensions and assumed quite different values from those acquired in the case of the original sets. This allowed rejection of the null hypothesis; stating that the analysed eye movement time series come from linear stochastic processes. 

### 4.2. Computation of Entropy

The second, important, nonlinearity measures—the correlation entropy *K_2_*—was evaluated with usage of the *sampleEntropy* function [41] taking the correlation sum, calculated in the previous step, as the parameter. Being interested in how the entropy changes during system evolvement, the set of measurements was divided into small groups representing characteristic phases of eye movement. The first of these periods is related to a change in a stimulus location that entails an eye movement to a new fixation. In order to determine this position and the velocity of a saccadic jump, the brain needs some time, therefore its reaction to a stimulus movement is delayed. This phenomenon is called *saccadic latency* and its duration depends on earlier-existing circumstances—it may amount to 90–120 ms, 135–170 ms or 200–220 ms [44]. Additionally, it frequently occurs that initially the saccade does not reach the fixation point, thus it must be corrected [45]. Then the eyes stay in a fixation, collecting information from the area of interest. This period may also differ in duration depend on the purpose of the fixation made. It may entail different eye movement characteristics. Taking all the above into account, several subsets for a time series were defined: <1…200>, <200…700>, <700…1500>, <1500…2999>. With the 1000 Hz sampling rate of measurement, elements in the subsets were separated by 1 ms. 

**Figure 8:**
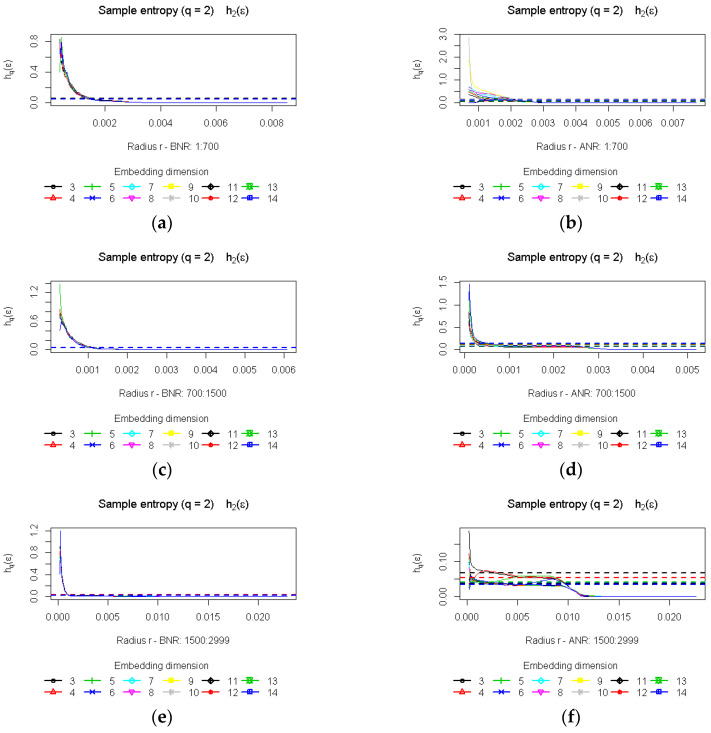
Plots of the entropy computed for: various embedding dimension, a given set of radiuses and three scopes: <1…700>—panels (**a**,**b**); <700…1500>—panels (**c**,**d**); <1500…2999>—panels (**e**,**f**). The BNR time series plots are shown in left-hand side panels while the ANR on the right.

The exploration of the two first scopes was difficult, or even infeasible, due to the limited number of elements in the majority of time series, consequently they were merged and finally three scopes were analysed <1…700>, <700…1500>, <1500…2999>. This procedure was applied to both types of time series: BNR and ANR, allowing more detailed analysis of the signal and identifying differences contributing to the observed patterns of behaviour in the time scale. Figure 8 presents sample charts of the entropy computed for: each embedding dimension and for a given set of radiuses; for one time series before (left-hand side) and after noise reduction (right-hand side); and for the three introduced scopes. In the first group of plots (BNR), only very small differences are visible when a different fixation part is taken into account and the entropy values turned out to be stable among varying embedding dimensions. The second, ANR time series exposed other outcomes, as some diversity is noticeable in the first and the third scopes for smaller radiuses. 

Similarly to the correlation dimension, the correlation entropy *K_2_* is determined based on a linear part—a scaling region—of entropy plots evaluated for a set of radiuses and embedding dimensions. Based on the results provided in Table 4, it may be noted that differences concerned the ANR time series in the first of the analysed scopes. The entropy was approximately 60% higher in comparison with the next two signal parts, where lower values—illustrating differences in the range of 10%—were obtained. The outcomes are different in the case of the BNR time series for which differences in the scope of 10–11% for all the analysed subsets were found. 

**Figure 9:**
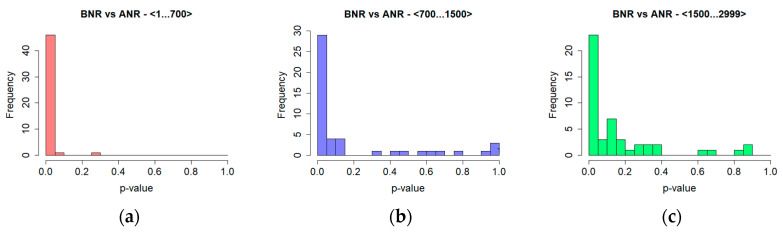
Significance of differences in the entropy values for time series BNR and ANR—histogram of *p*-values for various time series scopes (**a**) <1..700>, (**b**) <700…1500>, (**c**)<1500… 2999>.

The Shapiro-Wilk test revealed that the entropy values calculated for most cases were not normally distributed, thus the Wilcoxon test was used to verify the significance of differences in results. An examination was performed in regard to the BNF and ANR sets, as well as to all the scopes for the BNR and ANR time series independently. The results of the first test, which revealed a high percentage of significant differences in the first time series subset, are shown in the last row of Table 4 and in Figure 9. 

**Figure 10:**
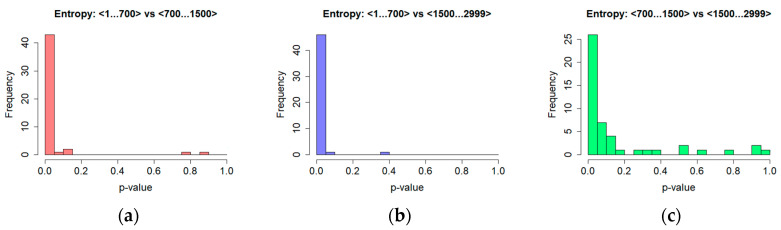
Significance of differences in the results obtained for the entropy for different parts of time series—histogram of *p*-values for ANR time series (**a**) <1…700> vs. <700…1500>; (**b**) <1…700> vs. <1500… 2999>; (**c**) <700…1500> vs. <1500… 2999>.

When the content of Table 5 and Figure 10 is analysed, it may be seen that the higher percentage of the participants’ sessions, which exposed significant differences in the evaluated entropy, concerned the ANR sets, especially when comparisons were made between the first and second, and the first and third scopes. It amounted to 90% and 96% of the sessions respectively. 

Once again, different results were obtained in the case of the BNR time series, which is represented by a lower percentage of statistically significant differences, particularly when comparison of the second and third scopes is considered. Only approximately 21% of participant’s sessions revealed such a difference in results. 

## 5. Discussion

Exploration of time series conducted with the usage of various methods of nonlinear time series analysis provided some premises, which allow perceiving eye movements as observed chaotic data. The first of them was revealed during the space-time separation plot analysis, applied to remove temporally correlated measurements. If a studied time series exposes chaotic behaviour, the contours in the plot initially rise and then oscillate around constant values. This feature was observed for both time series types: before and after noise reduction. The only observed difference concerned specific characteristics of oscillations, namely: their amplitude and frequency. According to [46] such behaviour can be associated with chaotic series. 

A subsequent argument was obtained based on the correlation dimension analysis, the method for quantitative assessment of a dynamic system nature. Lower values of this measure indicate a deterministic system, which additionally may be classified as chaotic if the correlation dimension is represented by a non-integer value. When studying the averaged results presented in Table 2, it may be noticed that for the three, differing in length, subsets of measurements—consisting of 1000, 1500 and 2999 elements—the correlation dimension assumed similar values, being lower than one. However, a slight increase of correlation dimension may be observed in the case of time series for which the filtering process was applied, especially in the case of the first observed scope (1000 points). 

Two conclusions may be drawn from this fact:

- the correlation dimension for eye movement time series may be reliably estimated when a set of measurements consists of 1000 and more samples, providing the opportunity to expand the possible scopes of the method application,
- the embedding dimension required—according to Taken’s theorem—to reconstruct a system’s phase space should at least be equal to 3 (*m* ≥ *2d* + 1) assuming *d* = 1 as the first integer greater than the estimated correlation dimension.

The second conclusion is in line with the results obtained in [21] where the embedding dimension evaluated in regard to the same set of time series—by means of the *False Nearest Neighbours* (FNN) method—mostly amounted to between 3 and 10. The values above 3 may be related to times series, for which the evaluated correlation dimension was greater than 1. Among the analysed ANR time series there were 22 such cases, and in this group, 14 with *D_2_* > 3; 5 with *D_2_* between 2 and 3; and 3 with *D_2_* between 1 and 2. These numbers could change a little if visual inspection of log(*C*(*r*)) versus log(*r*) plot were realised for each time series independently.

There were also some observations made when comparing the results obtained for the BNR and ANR time series. Although the averaged values of the correlation dimension belonged to a similar range, the analysis of correlation sum plots illustrated different distributions of these time series elements in the phase space. Elements of the first of the aforementioned sets were more scattered in comparison to the time series of the second type, which resulted in lower values of the correlation sum for small radius ranges. Moreover, this group of time series presented very similar patterns of log-log plots, which may suggest the existence of an additional component introduced by the recording setup. After removing this component, by means of the low-pass filter, similar plot patterns were visible in time series defined based on one person’s recordings, yet differed among participants. It means that noise elimination uncovered, during the fixation, more comparable eye movement velocities, which from time to time changed their characteristic towards higher values. 

**Figure 11:**
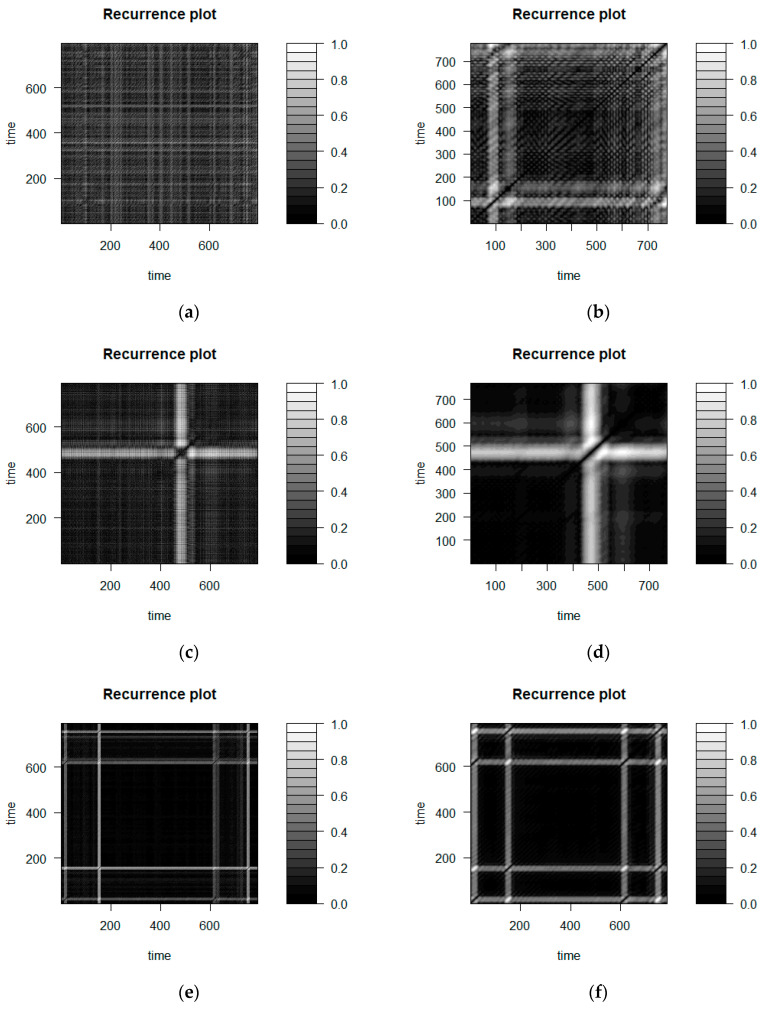
Recurrence plots for three sample time series, prepared for the subset of <700…1500> elements: panels (**a**,**c**,**e**) corresponds the BNR time series, while panels (**b**,**d**,**f**) to the ANR sets.

The existence of these differences may also be explored by means of the recurrence plot(Figure 11), a graph with time series elements located on axes X and Y according to their occurrence order. If *i*-th and *j*-th elements overlap each other, a point (*i*; *j*) is black on this plot. It is white when points are placed as far apart from each other as possible and shades of grey represent intermediate distances [47]. Figure 11a,c,e show recurrence plots for the three BNR time series in scope of <700…1500> elements, whereas Figure 11b,d,f contain the corresponding charts for the ANR sets. While comparing plots in the same row, it may be noticed that those obtained for the ANR time series have larger black areas; indicating smaller distances between chart elements, thus similar time series values; and wider white parts that represent elements with certainly greater distances from the rest of the points. The left-hand plots, although exposing similar general patterns, feature with less density, meaning that the smaller and larger distances between the values represented by the graph points occurred alternately. Additionally, the diagonal line which represents distances of points to themselves, is better visible in the right-hand plots. This provides another source of confirmation that neighbouring elements in the ANR time series are closer to each other than in the case of the BNR collections.

Finally, another argument for classifying eye movements as representatives of chaotic systems was gained when studying time series entropy. The initial state of an eye movement is determined by the point placed in some area in the phase space. Subsequently, during the system evolution, the area covered by the system increases. Therefore, if the initial state of the system was determined by a certain amount of information, more information is needed to describe the next states of the system. Sometimes this process is viewed as a loss of information, because the prediction of a system state based on the initial condition is less accurate [48]. The extent of this uncertainty is measured by the entropy, which in the case of chaotic systems assumes positive but finite value, while for random ones it tends towards the infinite. The lack of uncertainty is represented by zero value. In the case of complex biological systems which undergo evolution in a certain direction, the complexity grows over time and, in the process of their evolution, progressive “ordering” appears. The higher the level of system complexity, the lower the entropy

For both types of times series considered in this research, positive, yet limited, entropy was revealed—nonetheless of different characteristics. The entropy averaged over the time series before noise reduction (BNR) had similar values in the three analysed scopes and in many cases the existing differences turned out not to be significant. It indicates that systems along the whole registered period provided similar amount of new information regarding their states. This once again may be related to the existence of additional component in registered signals, introduced by the experimental setup. 

On the other hand, the time series after noise reduction exposed different entropy values in the different time series scopes. The highest value obtained for the first scope (Table 4), representing the first 700 ms of the registration time, points towards stronger chaotic behaviour when eyes initiate their reaction to a stimulus position change. Lower values presented for the further periods mean that, after starting from a chaotic movement, a system evolves in a more orderly manner. These outcomes confirm the findings described in [21,22], where chaotic behaviour was explored with the usage of the Largest Lyapunov Exponent. These studies revealed positive values of this exponent—corresponding to the chaotic signal nature—for all ANR time series in the scope of approximately 500 ms of the registration time and up to 700 ms for some of them. In the scope of 700–1500 ms, a signal’s characteristic fluctuating from convergent to chaotic and conversely. Such behaviour may be represented by positive entropy values—because one stable point was not reached—yet lower than for the first 700 ms.

## 6. Conclusions

The research presented in this paper was focused on eye movement analysis aimed at examining its nature in terms of the existence of nonlinear dynamics. Such nonlinear features with chaotic behaviour have been discovered in several biological systems. Among the studies conducted there are few investigating this issue in regard to eye movement signal, however this field has yet to be broadly explored. In these studies subsequent steps have been undertaken to extend knowledge in this area. Methods for nonlinear time series analysis: the fractal dimension and entropy were utilised for this purpose. The measurements obtained by means of the specialised set-up, based on the Jazz-Novo eye tracker, were used to define the time series; representing eye movement horizontal velocity. Although some methods’ parameters were approximated for the majority of the 1392 time series defined and the results were averaged over all, the conducted analysis of the eye movement enabled its classification as a signal exposing features characteristic of chaotic natures. However, it must be emphasised that obtaining confidence in differentiating chaotic and noise behaviours requires the application of various approaches. 

Eye movements are such kind of biological signals that may be influenced by memory, emotion or the ability to anticipate which may play an important role in system behaviour as well as by an experimental environment. Therefore, some pre-processing steps may be required before appropriate analysis commences. In the case of this research, the procedure of noise reduction was applied and the impact of the noise existence on the outcomes was explored. Although some significant differences were found, it has to be mentioned that there is a class of random noises which may give a finite estimate of *D*_2_ and a convergent *K*_2_ when finite-time series are considered [33]. This is an implication for verifying the research method used in regard to other eye movement data sets, planned as a future work. Furthermore, the application of a detailed analysis of recurrence plots in terms of chaos detection is considered [20,49]. 

Additionally, the results presented in this research were obtained for healthy, young people with normal vision, therefore, application of such studies for individuals affected by any abnormality in the eyes functioning or those suffering from brain diseases, would provide new insights into the usefulness of the presented method. Some steps were conducted towards this direction by the development of the platform for diagnosis and therapy of children with brain disabilities [50,51]. The continuation of these activities is also planned as future studies.

## Figures and Tables

**Table 1:** Limitation for evaluation of the correlation dimension given a set of measurements.

Number of Measurements	2log_10_*N*	Correlation Dimension
1000	6	≤6
1500	6.3522	≤7
2999	6.9539	≤7

**Table 2:** Averaged values of the correlation dimension evaluated for sets of three different lengths (1000, 1500, 2999) and for time series (TS) before and after noise reduction. The last row includes the percentage of users’ sessions revealing statistical significance of differences in the results.

TS Type	1000	1500	2999
BNR mean (stdev)	0.39 (0.18)	0.33 (0.14)	0.27 (0.09)
ANR mean (stdev)	0.49 (0.33)	0.35 (0.23)	0.24 (0.16)
BNR vs. ANR (*p* < 0.05)	67%	58%	65%

**Table 3:** Percentage of participants’ sessions exposing the statistical significance in differences in the estimated correlation dimension.

TS Type	1000 1500	1000 2999	1500 2999
BNR (*p* < 0.05)	41%	88%	60%
ANR (*p* < 0.05)	50%	90%	60%

**Table 4:** Averaged values of the entropy estimated for three intervals and for time series (TS) before and after noise reduction. The last row includes the percentage of users’ sessions exposing statistical significance of differences in the results.

TS Type	<1…700>	<700…1500>	<1500…2999>
BNR mean (stdev)	0.0480 (0.0141)	0.0588 (0.0148)	0.0542 (0.0203)
ANR mean (stdev)	0.1066 (0.0370)	0.0671 (0.0165)	0.0589 (0.0120)
BNR vs. ANR (*p* value <0.005)	95%	60%	47%

**Table 5:** Percentage of participant’ sessions revealing the statistical significance in the evaluated entropy.

TS Type	<1…700> <700…1500>	<1…700> <1500…2999>	<700…1500> <1500…2999>
BNR (*p* < 0.05)	63%	52%	21%
ANR (*p* < 0.05)	90%	96%	54%
